# A review on salivary constituents and their role in diagnostics

**DOI:** 10.6026/973206300181021

**Published:** 2022-10-31

**Authors:** Nishath Sayed Abdul, Sara Mohammed AlGhannam, Atheer Abdulaziz Almughaiseeb, Faisal Ahmed Bindawoad, Sara Mohammed alduraibi, Mahesh Shenoy

**Affiliations:** 1 Faculty of Oral and Maxillofacial Surgery and Diagnostic Sciences, College of Dentistry, Riyadh Elm University, Riyadh, KSA; 2 MOH, Prince Saud Bin Jalawi hospital, Alahsa, KSA; 3 MOH, Al-Muzahmiyah general hospital, Riyadh, KSA; 4 MOH, Thadiq general hospital, Riyadh, KSA; 5 Altakhassusi Hospital, Riyadh, KSA

**Keywords:** Biomarker, diagnostics, saliva, salivary constituents, systemic diseases

## Abstract

Salivary secretions possess a number of biomarkers beneficial for the diagnosis of a plethora of oral and other systemic disorders. Therefore, it is of interest to analyse and discuss the significance of saliva and its constituents as a valuable tool in aiding diagnostics in clinical settings by reviewing available literature and controlled trials. Using the PRISMA framework, a thorough review of research that were listed in the PubMed, Web of Science, and Cochrane library databases was performed which revealed 212 papers, 54 of which were thoroughly evaluated. Exclusion and inclusion criteria were used to choose studies that were applicable for the review. The selected studies reported a number of diseases that were diagnosed using salivary constituents in the same way as other methods of diagnosis, with a number of benefits. Thus, data shows that saliva is an excellent source for biomarkers which can be used for furthering medical diagnosis like other diagnostic procedures.

## Background:

Cancer, cardiovascular, metabolic, and neurological conditions are examples of human diseases with a global influence. The diagnosis of many medical problems is growing difficult, necessitating the addition of laboratory tests to clinical evaluation. [1] Salivary diagnostics has a lot of potential as an efficient method for early diagnosis, prognostication, and post-therapy status monitoring. The secretions of the major and minor salivary glands, mucosal transudations, gingival crevicular fluid, serum and blood from oral wounds, desquamated epithelial cells, expectorated bronchial and nasal secretions, bacteria and bacterial products, viruses and fungi, other cellular components, and food debris are all included in whole saliva. [2] A vast collection of hormones, proteins, enzymes,antibodies, antibacterial agents, and cytokines are all present in this complex fluid. Trans cellular, passive intracellular diffusion, active transport, or para cellular routes by extra cellular ultra filtration within the salivary glands or through the gingival crevice are the mechanisms by which these elements from the blood enter the saliva.[3] Saliva as a clinical tool has various benefits over serum and tissues, including non-invasive sample collection, smaller sample aliquots, strong patient compliance, cost effectiveness, simple storage and transit, higher sensitivity, and association with blood level measurements. Numerous salivary biomarkers, including those for cancer, autoimmune, viral, bacterial,cardiovascular, and metabolic illnesses, have been identified thanks to promising new technologies. [4] Numerous electrolytes, such as sodium, potassium, calcium, magnesium, bicarbonate, and phosphates, can be found in saliva. Immuno globulins, proteins,enzymes, mucins, and nitrogenous substances like urea and ammonia can also be detected in saliva. [5] These parts work in concert to provide the general functions listed below: Macromolecule proteins and mucins act to clean, aggregate, and/or attach oral microorganisms and contribute to the metabolism of dental plaque. [6] Calcium, phosphate, and proteins work together as an anti-solubility factor and modulate demineralization and remineralisation. Immuno globulins, proteins, and enzymes have an antibacterial effect. Bicarbonates, phosphates, and urea act to modulate pH and the buffering capacity of saliva. [7] Despite typically occurring in modest quantities and fluctuating with variations in flow, the components mentioned above continuously perform a variety of crucial tasks. It is crucial to emphasise that saliva must be viewed as a whole that is bigger than the sum of its parts because it is a special type of biologic fluid. Proteins in particular are multi functional, redundant, and amphi functional salivary constituents. [8] More than 99 percent of the fluid in saliva is water, making it a relatively diluted fluid. Plasma ultra filtrates do not include saliva. When saliva first forms in the acini, it is isotonic, but as it passes through the duct network, it becomes hypotonic. [9] Uns timulated saliva is hypotonic, which enables the taste buds to detect many flavours without being obscured by normal plasma sodium levels. Mucin glycoproteins, which cover and protect oral tissues, can expand and hydrate due to hypotonicity, particularly during low flow periods. [10] Un stimulated saliva has lower concentrations of glucose, bicarbonate, and urea, which contribute to the hypotonic environment and improve flavour. Saliva typically has a pH between 6 and 7, which indicates that it is mildly acidic. From 5.3 (low flow) to 7.8 (high flow), the pH of saliva can fluctuate (peak flow) [11]. While minor salivary glands supply little secretion volume and the majority of the blood-group components, major salivary glands contribute the majority of the secretion volume and electrolyte content to saliva.[12] In order to preserve dental health and establish a proper ecological balance, salivary function can be divided into five primary categories: lubrication and protection, buffering action and clearance, preservation of tooth integrity, antibacterial activity, and taste and digestion. [13] As previously mentioned, salivary components collaborate in overlapping, multifunctional roles that can be both advantageous and harmful. [14] Saliva acts as a barrier against irritants by lubricating and shielding oral tissues with a seromucous layer. Proteolytic and hydrolytic enzymes produced by plaque are among these irritants, along with possible carcinogens from smoking and exogenous chemicals and desiccation from mouth breathing. Mucins, which are secreted by small salivary glands, are the best lubricating substances in saliva. [15] Complex protein molecules known as mucins are primarily found in molecular weight categories and are made up of polypeptide chains that adhere to one another. These mucins exhibit great adhesiveness, high elasticity, high viscosity, and low solubility. [16] Any intra oral contact between soft tissues, between soft tissues and teeth, or between soft tissues and prosthetics is made more comfortable by saliva's lubricating properties, which are predominantly provided by these mucins. The lubricating properties of mucins facilitate mastication, speaking, and swallowing. [17] In addition to controlling bacterial and fungalcolonisation, mucins also serve an antibacterial purpose by moderating the adherence of microorganisms to the surfaces of oral tissue. A high-molecular-weight, highly glycosylated mucin (MG1) and a low-molecular-weight, single-glycosylated peptide chain mucin are both present in the sublingual and sub mandibular glands' secretions (MG2). [18] In the past 20 years, a lot of research has been centred on the significance of these two main mucins. As MG1 adheres firmly to the tooth, it helps to form the enamel pellicle, which guards the tooth against acid problems. Along with enticing some bacteria for attachment, MG1 produces heterotypic complexes with other salivary proteins such amylase, proline-rich proteins, statherin, and histatins. These complexes act as a temporary food source for bacteria [19]. MG2 adheres to enamel but is quickly dislodged. It encourages oral bacteria, such as Streptococci mutans, to aggregate and be removed from the body. Patients who are resistant to caries have more MG2 in their saliva than those who are vulnerable to the disease. [20] The ability of salivary mucins to control intercellular calcium levels is a crucial aspect of their multifunctional role in maintaining mucosal integrity. By encouraging the development of healthy commensal oral flora, creating a barrier of protection and lubrication against excessive wear, acting as a diffusion barrier against acid penetration, and regulating mineral egress from the tooth surface, mucins, which are a component of the enamel pellicle, aid in the initialization of bacterial colonisation. According to research findings, salivary mucins carry out a number of vital tasks for preserving a strong mouth defence. [21] A second purpose of saliva is buffering and clearing through the following elements: Amphoteric proteins and enzymes, as well as bicarbonate, phosphate, and urea [22]. The most crucial buffering mechanism is bicarbonate. By neutralising acids, it diffuses into plaque and serves as a buffer. Additionally, it produces ammonia, which is then converted into amines, which act as a buffer by neutralising acids. Low-molecular-weight, histidine-rich peptides are responsible for more than 90% of saliva's non-bicarbonate buffering capacity. Another buffer found in saliva, urea, causes plaque to metabolise ammonia, which raises the pH of the plaque [23]. During periods of low flow with unstimulated saliva, the buffering effect is essentially non-existent, while it is more effective during stimulated high flow rates. Only during unstimulated flow is phosphate expected to be significant as a buffer. [23] The replacement of missing minerals takes place through the organic enamel matrix to the crystals. Mineral super saturation in saliva is essential to this process. The development and remineralization of enamel may be attributed to the high levels of calcium and phosphate in saliva that are maintained by salivary proteins. Salivary peptide statherin, which binds to hydroxyapatite, helps to stabilise the calcium and phosphate salts solution and acts as a lubricant to shield the tooth from wear [24]. It may also help to start the production of the protective pellicle. Statherin, histatins, cystatins, and proline-rich proteins, which are present in the protective pellicle, are too big to enter the pores of the enamel. Because they allow minerals to enter the enamel for remineralization and limit mineral outflow, they stay on the surface, bound to hydroxyapatite, to help control the crystalline development of the enamel. The stability of hydroxyapatite in the outer tooth structure is improved by this control of precipitation and mineral outflow. Dental plaque fluid is believed to trade low-molecular-weight protein fractions, which are assumed to be produced by the proteolytic degradation of bigger proteins. At the tooth-saliva interfaces, these protein fractions assist regulate and enhance remineralization, microbial adhesion, and plaque metabolism. [25] Fluoride in saliva accelerates the crystallisation process, resulting in a fluorapatite-like coating that is more resistant to caries than the original tooth structure. Because the enamel's magnesium and carbonate components are replaced by the more robust, caries-resistant fluor-apatite crystals, it has been hypothesised that small quantities of demineralization are beneficial for the tooth. Apatite crystals are prevented from dissolving by fluoride in saliva. [26] Given that saliva plays a role in the demineralization-remineralization process, it's crucial to keep an eye on salivary flow, particularly in patients who are taking numerous drugs or who have systemic conditions that reduce salivary flow. [27] Fluoride administration can encourage remineralization in patients with exposed root surfaces, recurrent carious lesions, or incipient lesions. For patients with salivary hypofunction, salivary stimulants and replacements should also be encouraged. A technique for genetically engineering salivary proteins and other salivary components is currently being researched for application in future artificial salivas [28]. Because plaque and other food item tenaciously cling to hard and soft tissue surfaces in typically dry conditions, home care for people with decreased salivary flow becomes a time-consuming operation. Due to tissue desiccation and the ensuing difficulty in manipulating tools and materials in such circumstances, even professional therapy for individuals with severe salivary dysfunction presents a problem. Clinicians should resist the urge to "overexplore" lesions with white spots. The area's ability to remineralize may be hampered by excessive manipulation of the crystalline structure. [29] Saliva's antimicrobial properties are a fourth function. The exocrine salivary glands protect teeth and mucosal surfaces by secreting fluid that contains immunologic and non immunologic substances. Saliva contains secretory IgA, IgG, and IgM, which are immunologic components [30]. Selected proteins, mucins, peptides, and enzymes make up the non immunologic salivary contents. The majority of the immune system's components found in saliva, including secretory IgA, are produced by plasma cells in connective tissues and transported by duct cells from the major and minor salivary glands. While present on mucosal surfaces, IgA also functions as an antibody to bacterial antigens, neutralises viruses, and aggregates or clumps bacteria to prevent them from adhering to host tissues. Low levels of other immunoglobulins are also seen in saliva and they most likely originate from gingival crevicular fluid. It appears improbable that the oral fluid would be affected generally by host complement reaction. IgA by itself does not activate complement; however when gingivitis is present around existing teeth, gingival crevicular fluid host complement components can enhance oral fluids. [30, 31] Specifically, plasma and ductal cells, which have varied reactions to stimulation and differing content levels, are the two separate sources of immunologic and nonimmunologic antibacterial salivary content. Proteins, mucins, peptides, and enzymes (lactoferrin, lysozyme, and peroxidase), all made by acinar gland cells, are nonimmunologic antibacterial salivary components that aid in defending teeth against microbial, chemical, and physical harm. [32] More firmly than either MG2 or IgA by itself, the low-molecular-weight mucin and IgA complex bind mucosal microorganisms. Intercalated ductal cells generate lactoferrin, which binds ferric iron in saliva. Microbes that require ferric iron as a food supply, like cariogenic streptococci, are prevented from using it by this procedure. Nutritional immunity refers to the process of depriving bacteria of essential nutrients. Because Streptococcus mutans is sensitive to lactoferrin, it also has an additional antibacterial impact unrelated to its iron-binding capacity. [33] In order to destroy and stop bacterial development, lysozymes, which are produced by the basal cells of striated ducts in parotid glands, crack bacterial cell walls. Additionally, lysozymes encourage the aggregation of bacteria to clear them out. [34] Lysozymes from plasma are also present in gingival crevicular fluid. Peroxidase sometimes referred to as sialoperoxidase or lactoperoxidase, catalyses the conversion of bacterial metabolic waste products into the highly poisonous thiocynate. Peroxidase, which is secreted by acinar cells, also shields mucosa from the potent oxidising effects of hydrogen peroxide created by oral bacteria. A little part in controlling salivary calcium is played by the family of proteins known as cystatins. [35] However, it's possible that the primary function of cystatins is to block the cysteine-proteinase responsible for the pathogenesis of periodontal disease. Finally, proteins that assemble bacteria include glycoproteins, statherins, agglutinins, histadine- and proline-rich proteins. This "clumping" process, which was previously discussed, decreases bacteria's capacity to stick to intraoral surfaces made of hard or soft tissue, which inhibits the growth of bacteria, fungi, and viruses. Protein content generally rises in direct proportion to flow rate. However, salivary protein concentrations may be influenced by stress, inflammation, infections, and hormone changes, as well as by circadian oscillations, as do other salivary components. Protein content also differs between individuals, displays various polymorphic behaviours, and can show strain-species differences in protein-microbial interactions. [36] The idea that saliva has antimicrobial properties emphasises the therapeutic benefit of promoting natural saliva, particularly in individuals with impaired function. Saliva substitutes are very beneficial for dental hygiene, tooth integrity, and lubrication, but they don't provide much protection that can match that provided by natural salivary components. The creation of efficient artificial saliva is a challenging endeavour since salivary components are thought to be multifunctional (i.e., having "built-in" compensatory redundant antibacterial activities) and amphi functional, depending on the intraoral environment or the molecule. [37] Enhancing flavour and starting the digestive process are saliva's final and fifth functions. Saliva's hypotonicity improves the ability to taste salty meals and nutrient sources. Protein and gustin, which bind zinc, are necessary for this improved taste perception. Amylase is a significant component of parotid saliva that initially dissolves sugar, starts the breakdown of starch, giving saliva an early but limited function in overall digestion. Because pancreatic amylase, rather than salivary amylase, is responsible for the majority of the digestion of starch, saliva plays a relatively small role in starch breakdown. Fat digestion is also started by salivary enzymes. More importantly, saliva facilitates swallowing by lubricating the meal bolus. It seems obvious that artificial supplements would be challenging to make when one considers the role of saliva in taste and early digestion. [38]

## Materials and Methods:

## Study Design:

This present systematic review was carried out with a search in the literature that includes original full text articles, cross-sectional, observational, descriptive studies, those published from June 2007- June 2022.This study was be registered with the research center of Riyadh Elm University for institution review board approval with registration number FRP/2022/454/781.

## Protocol employed:

This systematic review was performed as per the Preferred Reporting Items for Systematic Reviews and Meta-Analysis (PRISMA) strategy and rules from the Cochrane Group and the book Orderly Reviews in Health Care: Meta-examination.

##  Study hypothesis:

Saliva/salivary constituents have been known to be a source of multiple biomarkers. Hence, we aim to analyse through this systematic review whether it performs on the same level as other methods of diagnosis.

## Eligibility criteria:

For this systematic review, only papers published in English were considered. The analysis of the significance of saliva/salivary constituents in clinical diagnostics was the primary objective of this review, so as such we avoided any studies where the diagnosis methods involved employment of saliva as a preliminary form of testing where the results can be ambiguous or are dependent upon a number of variables which might or might not be under the control of the investigator; for example, the rapid diagnostic kit (RDT) which is used for the preliminary detection of COVID-19 has a tendency to give false negative reports. Also, we ensured the studies that were selected for the meta-analysis included full text articles and had a substantial sample size in order to verify the credibility of the study. Though the literature available on this topic is quite substantial in volume, we didn't limit our search in terms of the time period when the studies were published i.e., we took into account all the papers that were published with context to our topic (where the number of papers itself was found to be sparse). Placebo-controlled studies were not included in the analysis. Case studies, case series, and other non-randomized trials were also omitted. Excluded were animal studies, literature reviews, and cases published in languages other than English.

## Study selection:

There were a total of 212 documents showed after extensive search on the online journals and 109 of the papers were selected initially. Following that, 61 similar/duplicate articles were eliminated, which resultantly made 48 separate papers available at first. A further 29 papers were screened out of these papers after further scrutiny, which made 19 articles available for our review. The abstracts and titles of submissions were then reviewed, and a further 13 papers were eliminated. Finally, 6 documents that met the inclusion and exclusion criteria were chosen, which included study articles and randomised control trials.

##  Search strategy:

The following databases were scoured for studies pertaining to the incidence of atypical tuberculosis in various organs: MEDLINE, PsychINFO, PubMed, Indian Council of Medical Research, and Cochrane. Other published works were found by scanning the reference lists of pertinent papers.

## Results:

The results of the systematic review have been tabulated in table 1 presented below, with the details of the 6 studies that were selected for the review presented in the table, following which the Figures 2, 3 and 4(see PDF) given below shows the results of the meta-analysis (using RevMan 5 software) in the form of a forest plot depicting all the studies taken up in this systematic review and evaluating them as comparative scenarios between the clinical trial group vs. control groups. Results of the meta-analysis clearly reveal that all the selected studies support our initial hypothesis that salivary constituents act as viable biomarkers performing on the same parlance as other diagnostic methods. The viability is further demonstrated by the fact that the disorders we analysed in the studies are generally systemic in nature, for example OSCC is known to be notorious in terms of its prognosis as well as the remission phase.

## Discussion:

Reduced salivary and lacrimal gland secretion as well as endocrine dysfunction is two features of the autoimmune illness Sjogren's syndrome (SS). Analysis of salivary secretions (also referred to as sialo chemistry) is quite useful for SS diagnosis. Sjogren's syndrome is characterised by a rise in the levels of immunoglobulins, inflammatory mediators, albumin, sodium, and chloride and a reduction in the levels of phosphate. Lactoferrin, beta 2 microglobulin, lysozyme C, and cystatin C were found in higher concentrations in the salivary proteins study. However, salivary carbonic anhydrase and amylase levels were down. [39] Myocardial infarctions with ST-elevation, non-ST-elevation, and unstable angina are all included in the group of clinical syndromes known as acute coronary syndromes (ACS). [40] Atherosclerotic plaques that rupture and induce clinical symptoms like chest discomfort or acute myocardial infarction are what define it (AMI). Inflammation goes hand in hand with the atherosclerotic process, which is important key event of endothelial damage. C-reactive protein (CRP), myoglobin (MYO), creatinine kinase myocardial band (CKMB), cardiac troponins, and myeloperoxidase are salivary markers of cardiovascular diseases. [41] When combined with an ECG, these markers show a positive correlation with myocardial infarct patients as compared to healthy controls. Within 48 hours of the onset of chest discomfort in AMI patients, salivary MYO levels are noticeably increased. Additionally, MYO serum concentrations and salivary levels are favourably associated. Although CK-MB and troponins can be found in saliva, their diagnostic value is limited. In a study by Miller et al. they showed that patients with AMI had considerably greater salivary concentrations of CRP and TNF-, and that these salivary concentrations directly linked with the serum concentrations. [42] Additionally, it was discovered that AMI patients had higher levels of salivary myelo peroxidase. According to studies, patients with AMI have much higher levels of salivary soluble ICAM-1 than they do of salivary soluble CD40 ligand. An early stage of cardiovascular problems, hypertension, has been linked to higher amounts of salivary lysozyme. [43] Monitoring the quantity of oral bacteria in saliva is another use for it. Increased caries prevalence and root caries have been linked to increased levels of Streptococcus mutans and lactobacilli in saliva. Aspartate amino transferase (AST) and alkaline phosphatase levels have been linked to periodontal disorders (ALP). Periodontal disease can be tracked using salivary AST as a marker. Diabetes and periodontitis were linked to lower salivary uric acid and albumin levels. This might be explained by the oxidative stress that exists in the mouth cavity under these circumstances. [44]

Due to its usage in drug monitoring and the detection of illicit drugs, saliva has become increasingly important. Nicotine, cannabinoids, cocaine, phencyclidine, opioids, barbiturates, diazepines, amphetamines, and ethanol can all be found in saliva. [45] Only the unbound portion of the medication in the serum diffuses into the saliva during drug level monitoring and can be found there. The assessment of illicit drug usage is the most significant diagnostic use of saliva. The drug appears in the saliva for the same amount of time as it does in the serum; therefore its simple existence is sufficient for forensic reasons. [46] Tobacco smoke exposure can be tracked using salivary nicotine levels. Cotinine, the primary metabolite of nicotine, has been demonstrated to be a marker for both active and passive smoking. A hydrophobic porous silicon array can directly analyse saliva for metha mphetamine, cocaine, and 3,4-methyl enedioxy methamphetamine to detect illicit drug use quickly. Forensic toxicologists have had issues with drug misuse because of the endogenous nature of the – hydroxy butyric acid (GHB) in the blood and urine. Because of its advantages of simple, non-invasive collection and drug stability, saliva is a biological matrix utilised for drug testing of GHB levels. Additionally, the drug's levels in saliva and blood are correlated. [47]

 Forensics has frequently used salivary analysis. Glasses, cigarettes, food items, envelopes, and other items make it simple to collect salivary samples. Most patients release blood group antigens into their saliva, which can be used to identify criminal suspects and establish paternity in legal proceedings. Since DNA is very durable in a dry environment, salivary samples can be used for DNA testing. In cases of sexual abuse and harassment, genetic profiling can be used to identify DNA in saliva. The victim's saliva typically contains foreign DNA for up to 60 minutes, making it an important piece of forensic evidence. [48] In almost all kinds of cancer, early identification is essential for a good prognosis. Oral squamous cell carcinoma (OSCC) has been diagnosed using saliva as a diagnostic tool and salivary analytes like proteins, mRNA, and DNA. [49] Lung, breast, and prostate carcinomas are linked to aberrant expressions of long non-coding RNA (lncRNA). A study showed that saliva contains measurable levels of lncRNA, which may serve as OSCC indicators. Numerous disorders have been linked to deregulation of miRNAs, which are small non coding RNA molecules. [50] Patients with OSCC have miRNAs in their saliva, which might be utilised to diagnose them in addition to other methods. The non-invasive and affordable diagnosis of lung cancer can be aided by salivary mRNA biomarkers (CCNI, EGFR, FGF19, FRS2, and GREB1). AGPAT1, B2M, BASP2, IER3, and IL1B are the salivary mRNA biomarkers for ovarian cancer detection. [51] A tumour suppressor protein called p53 is created by cells in response to various forms of DNA damage. One of the main factors contributing to the onset of human cancer is the inactivation of the p53 protein during mutation. Patients with OSCC can have p53 antibodies found in their serum and saliva. Saliva has significantly higher levels of fibroblast growth factor 2 (FGF2) and fibroblast growth factor receptor 1 (FGFR1) in patients with salivary gland tumours, making it a possible biomarker for the early detection of salivary gland cancers. A recognised indicator of prostate cancer is prostate specific antigen (PSA) (PA). [52] In people with PA, there is a correlation between salivary and blood PSA levels. This may be an effective biomarker for PA. Plasma and saliva from OSCC patients were shown to have considerably higher amounts of salivary cortisol. This implies that this hormone may be employed as a clinical stage marker. OSCC has greater amounts of salivary lactate dehydrogenase, which makes it a potential marker. Patients with oral cancer had higher than normal amounts of salivary nitrate and nitrite. Squamous cell carcinoma of the tongue exhibits a gradual rise in salivary adenosine deaminase (ADA) activity from stage I to stage III. This biomarker might help with an early tongue SCC diagnosis. [53]

## Conclusion:

For a very long time, saliva has been considered to be a crucial diagnostic fluid. Salivary diagnostics are increasingly being used in clinical settings as a result of the increased efficiency of genomic and proteomic technologies in recent years. A recent development in the field of salivary diagnostics is called salivary metabolomics, which examines a wide range of low molecular weight endogenous metabolites found in saliva to identify disorders. The discovery of disease biomarkers and their translation from the laboratory to the clinical setting represent the most significant achievements in salivary diagnostics. However, the development of salivary diagnostics has been hampered by a lack of sensitive detection techniques, a lack of connection between blood and salivary macromolecules, and diurnal fluctuations in saliva. Salivary diagnostics, on the other hand, offers a simple, affordable, painless, and stress-free method of illness diagnosis in contrast to blood and other bodily fluids. Recognizing the numerous contributions saliva provides to the preservation and maintenance of dental and systemic health is important, regardless of whether it is produced in great or small amounts.

## Readers' comments:

The authors did not cite the following article Malathi N, Mythili S, Vasanthi HR. Salivary diagnostics: a brief review. ISRN Dent. 2014 Jan 29; 2014:158786. doi: 10.1155/2014/158786. PMID: 24616813; PMCID: PMC3926256. https://pubmed.ncbi.nlm.nih.gov/24616813/
